# Therapeutic Notes

**Published:** 1913-08

**Authors:** 


### Therapeutic Notes.

Uric acid, put up in ampoules with adrenailin and cocaine, is advised by Rosenberg of Berlin, for gout, with the idea of producing antibodies which shall stimulate phagocytosis.

Salvarsan, 1 part; water, 20; glycerin up to 150, has been successful as a swab treatment for old syphilitic glossitis by Allport of England.

Oil of cinnamon, cloves and peppermint, 2/3 c.c. in capsule, iv. i. d., each oil being used for a day in succession, is the suggestion for the treatment of coryza, by French of Woolwich.

Ivy poisoning is said to be relieved by the juice of the leaves of impatiens fulva, snap dragon, or jewel weed, sometimes erroneously called lady slipper. This is a common plant of shady, damp places, found near poison ivy and hence tending to corroborate the old idea that the antidote of a poison was always at hand.

The arrow poison of the Columbian (South America) Indians has been found by W. Casper and A. Loewy, to consist of the secretion of a small frog. It paralyses the animal, which is then killed. The flesh is not poisonous. The ordinary edible frog, rana esculenta, is said to produce a similar poison. It is possible that this may contain a valuable therapeutic suggestion.

For hyperidrosis, sponging with 1-2 per cent. of formalin solution or a dusting powder of 1 part of salicylic to 8 parts of boric acid, is recommended. Six milligram doses of agaricin, repeated every hour for three doses may be used if local measures fail. To neutralize the odor of perspiration, especially in the axillae and perineum, a saturated solution of sodium bicarbonate, which may also be allowed to dry upon garments, is efficient.

For third degree burns, Ohleyer recommends a thick covering of magnesium carbonate, the adherent crusts being removed in dressings, with 1:100 solutions of lysol.

Major H. C. French of England, advises freezing chancroids with ethyl chlorid spray, making three applications and then dressing with iodoform.

Kolipinski claims good results from 1-2 per cent. solutions of nickel sulphate applied on compresses, for alopecia areata.

Warner's Safe Cure is said to consist of fluid extract of gaultheria 2 ounces, same of gentian, sarsaparilla, taraxacum, calumbo, each one ounce; same of podophyllum and buchu, each one-half ounce; spirits of juniper 1 pint; white sugar ¼ pound.

